# Case-control genome-wide association study of rheumatoid arthritis from Genetic Analysis Workshop 16 using penalized orthogonal-components regression-linear discriminant analysis

**DOI:** 10.1186/1753-6561-3-s7-s17

**Published:** 2009-12-15

**Authors:** Min Zhang, Yanzhu Lin, Libo Wang, Vitara Pungpapong, James C Fleet, Dabao Zhang

**Affiliations:** 1 Department of Statistics, Purdue University, 150 North University Street, West Lafayette, IN 47907, USA; 2 Department of Foods and Nutrition, Purdue University, 700 West State Street, West Lafayette, IN 47907, USA

## Abstract

Currently, genome-wide association studies (GWAS) are conducted by collecting a massive number of SNPs (i.e., large *p*) for a relatively small number of individuals (i.e., small *n*) and associations are made between clinical phenotypes and genetic variation one single-nucleotide polymorphism (SNP) at a time. Univariate association approaches like this ignore the linkage disequilibrium between SNPs in regions of low recombination. This results in a low reliability of candidate gene identification. Here we propose to improve the case-control GWAS approach by implementing linear discriminant analysis (LDA) through a penalized orthogonal-components regression (POCRE), a newly developed variable selection method for large *p *small *n *data. The proposed POCRE-LDA method was applied to the Genetic Analysis Workshop 16 case-control data for rheumatoid arthritis (RA). In addition to the two regions on chromosomes 6 and 9 previously associated with RA by GWAS, we identified SNPs on chromosomes 10 and 18 as potential candidates for further investigation.

## Background

Genome-wide association studies (GWAS) are challenged by the "curse of dimensionality", i.e., a large number of single-nucleotide polymorphisms (SNPs) are genotyped (i.e., large *p*) from a small number of biological samples (i.e., small *n*). Because of this, in practice, only one SNP is evaluated for association at a time [1]. However, such univariate approaches ignore the high correlation between SNPs in certain regions of the genome due to linkage disequilibrium (LD) [2]. Recently, Zhang et al. [3] developed a penalized orthogonal-components regression (POCRE) method for efficiently selecting variables in large *p *small *n *settings. Here we propose to implement linear discriminant analysis (LDA) combined with POCRE, and apply the so-called POCRE-LDA to a case-control GWAS dataset.

## Methods

### POCRE

POCRE works well to fit a large *p *small *n *regression model [3],

where the sample (**Y**, **X**_**1**_,..., **X**_**p**_) is of size *n*. Let , and further assume both **Y **and **X **are centralized (*μ *= 0 in the above model). Starting with , POCRE sequentially constructs components  such that  is orthogonal to , and the loading *ω*_*k *_= *γ*/||*γ*|| with *γ *minimizing

Here *g*_*λ*_(*γ*), is a penalty function with tuning parameter *λ*, which Zhang et al. [3] implemented with empirical Bayes thresholding methods proposed by Johnstone and Silverman [4]. Such implementation introduces a proper regularization on *γ*, and provides adaptively sparse loadings of orthogonal components.

When the optimal *γ *solving Eq. (2) is zero, we stop the sequential construction because the constructed orthogonal components  account for almost all contributions of **X **to the variation in **Y**. An estimate of *β*_1_,⋯, *β*_*p *_in Eq. (1) can be derived by regressing **Y**on these orthogonal components. Resultant estimates of *β*_1_,⋯, *β*_*p *_are mostly zero due to the sparse loadings in *ω*_*j*_, *j *= 1, 2,⋯. This algorithm is computationally efficient as it only involves constructing penalized leading principal components.

### POCRE-LDA

POCRE can efficiently construct orthogonal components by excluding insignificant SNPs, and therefore simultaneously identify significant SNPs for GWAS [5]. In a case-control GWAS, we can define the response variable using the group membership, i.e., *y*_*i *_= 1 if individual *i *is from the case population, and *y*_*i *_= -1 otherwise. Then, regressing **Y **= (*y*_1_,⋯, *y*_*n*_)^*T *^on **X **using POCRE implements LDA with threshold *c *= 0. Indeed, the resultant  is a penalized version of Fisher's LDA direction [6], with *b*_*j *_estimating *β*_*j*_. We therefore call it POCRE-LDA, with the tuning parameter *λ *elicited by employing a 10-fold cross-validation and considering candidates *λ *∈ {0.8, 0.82, 0.84, 0.86, 0.88, 0.9, 0.92, 0.94, 0.96, 0.98, 1}.

We applied POCRE-LDA to the rheumatoid arthritis (RA) case-control data in Genetic Analysis Workshop (GAW) 16. Of the 545,080 SNPs, 490,613 (90.2%) SNPs and all 2,062 individuals (868 cases and 1,194 controls) were kept for our analysis after using PLINK [7] to preprocess the data and control the data quality. To control the underlying population structure, EIGENSTRAT [8] was used to derive the first 10 principal components of the genome-wide genotype data. Then POCRE-LDA was applied separately to each chromosome. The effects of the 10 principal components constructed by EIGENSTRAT were controlled, where, for each chromosome, only the first several principal components were identified to be associated with the case/control status (results not shown).

## Results

**Figure 1:**
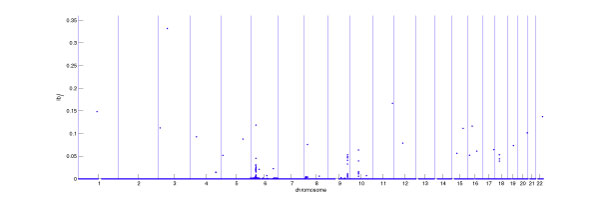
**SNPs identified using POCRE-LDA**. The *x*-axis indicates the physical location of each SNP on the chromosome, and the *y*-axis represents the absolute value of the estimated coefficient, i.e., |*b*_*j*_|. Genetic regions with multiple SNPs are identified in chromosomes 6, 8, 9, 10, and 18.

The results of our analysis are shown in Figure 1, where the estimated effect size of each SNP is plotted against the physical location of the SNP. Several clusters of nonzero effects appear on chromosomes 6, 9, 10, and 18. The cluster on chromosome 6 covers a wide genomic region ranging from 6p22.1 to 6p21.32 and includes many genes related to the immune system. For example, this region contains the human leukocyte antigen (HLA) genes that encode the major histocompatibility complex (MHC) proteins necessary for antigen presentation and the *TAP2 *gene that encodes a membrane-associated ATP-binding cassette peptide transporter necessary for delivering antigens to MHC class I proteins. Because there are many SNPs with nonzero effects in each of these clusters, Table 1 reports only the most significant SNPs within each region. The gene information corresponding to this genetic location was obtained from the Ensemble database http://www.ensembl.org. Several of the genes on chromosome 6 listed in Table 1 have previously been shown to be associated with RA, i.e., *MICB *[9], *BAT1 *[9], *TAP2 *[10,11], and *BTNL2 *[12]. The most significant SNP on chromosome 9 (rs2900180), together with another significant SNP in that region (rs3761847), are in LD with the TNF receptor associated factor 1 (*TRAF1*) gene as well as the *C5 *gene. Polymorphisms in these two genes were previously associated with RA [13,14]. Our results suggest weak evidence of association for SNPs on chromosomes 10 and 18 with RA (i.e., only a few SNPs with nonzero effects occur there). Neither of these regions has previously been associated with RA. Our analysis also reveals a large number of individual SNPs with nonzero effects (Figure 1). These may also reflect genetic variation controlling the risk for RA. For example, rs2476601 on chromosome 1 has a nonzero effect and is about 71 kb upstream of the *PTPN22 *gene identified by Plenge et al. [14] as associated with RA.

**Table 1:** Genomic regions and candidate genes identified for case-control study of rheumatoid arthritis in GAW16

Chromosome	Genomic region (Mb)	SNP with the largest effect	Number of genes	Candidate genes
6p21.33	31.55-31.62	rs2523647	4	*MICB-001*; *MICB-002*; *MCCD1-001*; *BAT1*
6p21.32	32.33-32.41	rs10484560	1	*C6orf10*
6p21.32	32.47-32.69	rs3135363	6	*BTNL2*; *HLA-DRA*; *AL662796.6*; *HLA-DRB9*; *HLA-DRB5; HLA-DRB1*
6p21.32	37.74-32.79	rs9275601	1	*HLA-DQB1*
6p21.32	32.87-32.97	rs9380326	5	*HLA-DOB*; *TAP2*; *PSMB8*; *PSMB9*; *TAP1*
6p21.32	33.21-33.29	rs3130237 rs6901221	5	*COL11A2*; *RXRB*; *SLC39A7*; *HSD17B8*; *RING1*
9q33.1	12.05-12.12	rs2900180	3	*DBC1*; *TRAF1*^a^; *C5*^a^
10q11.22	49.64-49.79	rs2671692	2	*C10orf64*; *LRRC18*
18q12.1	26.82-26.88	rs2852003	1	*DSC3*

^a^The identified SNPs are in linkage disequilibrium with these genes.

## Discussion

For general settings with large *p *small *n *data, the superior performance of POCRE over existing methods such as LASSO and ridge regression were presented in Zhang et al. [3]. The results of our analysis compare favorably with the earlier GWAS conducted by Plenge et al. [14]. This is in spite of the fact that we conducted only a stage I analysis (i.e., a full GWAS in a single population) rather than the two-stage approach reported by Plenge et al. [14] (i.e., follow-up analysis of a sub-set of highly significant SNPs identified from stage I using a second, unrelated population). Thus, our new analytical procedure appears to be more sensitive and less open to false positives compared with the traditional univariate approach used by Plenge et al. [14]. In addition to the multi-SNP approach we used, another difference between our approach and the method used by Plenge et al. [14] is that we used the first 10 principal components for population stratification in our analysis, whereas Plenge et al. [14] used only the first principal component. It should be noted that our analysis did not find an association between the *STAT4 *gene polymorphism and RA that was previously reported by others [15]. However, this earlier analysis was conducted in a case-control association study using only 13 candidate genes selected from within the long (q) arm of chromosome 2 that was previously shown to be in linkage with RA in 642 families of European ancestry [15]. Our data showing a lack of association between the *STAT4 *polymorphism and RA is consistent with the previous GWAS by Plenge et al. [14].

## Conclusion

Combination of the novel method POCRE with LDA allows us to identify genomic regions (chromosomes 6, 9, 10, and 18) harboring genes associated with the susceptibility to RA. In addition, we identified several single SNPs that are in LD with genes that have previously been associated with RA.

## List of abbreviations used

GAW: Genetic Analysis Workshop; GWAS: Genome-wide association studies; HLA: Human leukocyte antigen; LD: Linkage disequilibrium; LDA: Linear discriminant analysis; MHC: Major histocompatibility complex; POCRE: Penalized orthogonal-components regression; RA: Rheumatoid arthritis; SNP: Single-nucleotide polymorphism

## Competing interests

The authors declare that they have no competing interests.

## Authors' contributions

MZ and DZ both conceived the study and drafted the manuscript. MZ and YL designed the study and performed statistical analysis. LW and VP participated in the design of the study and preprocessing of the data. JCF participated in interpreting the statistical analysis results, reviewing and editing the manuscript. All authors read and approved the final manuscript.
